# Breast Cancer Clinical Trials: The Landscape at the Uganda Cancer Institute and Lessons Learned

**DOI:** 10.1200/GO.20.00185

**Published:** 2021-01-15

**Authors:** Manoj P. Menon, Nixon Niyonzima, Julie Gralow, Jackson Orem

**Affiliations:** ^1^ Fred Hutchinson Cancer Research Center, Seattle, WA; ^2^ University of Washington, Seattle, WA; ^3^ Seattle Cancer Care Alliance, Seattle, WA; ^4^ Uganda Cancer Institute, Kampala, Uganda

## Abstract

The Uganda Cancer Institute, the sole national comprehensive cancer center in Uganda, has a long and rich history of clinical investigation and locally relevant cancer research. Given the increasing burden of breast cancer in Uganda and elsewhere in sub-Saharan Africa (SSA) and driven by the limited availability of immunohistochemistry (IHC), we launched a clinical trial aimed at evaluating locally available diagnostics to detect the presence of hormone receptors (estrogen receptor and progesterone receptor) and human epidermal growth factor receptor 2. Preliminary data from 32 women in the diagnostic component of the study reveal high sensitivity and specificity for estrogen receptor and progesterone receptor and high specificity for human epidermal growth factor receptor 2 when comparing reverse transcriptase polymerase chain reaction with the gold standard (IHC). Innovative diagnostic and treatment strategies are required to address the burden of breast cancer that is increasing throughout SSA. Given the costs, infrastructure, and trained personnel associated with IHC, alternative testing options (including reverse transcriptase polymerase chain reaction as tested in our study) may provide an expedited and cost-effective method to determine receptor testing in breast cancer. Clinical trials conducted in the local setting are critical to determining optimal strategies for effective breast cancer management in SSA.

## BACKGROUND

Cancer is an emerging health threat in resource-limited countries, including sub-Saharan Africa (SSA), where its morbidity and mortality are expected to exceed the burden in resource-rich regions in 2020.^1^ Multiple factors, including advanced stage at presentation, limited diagnostic capacity, therapeutic options, and access to clinical trials and novel therapies are responsible for this disparity.^2-8^

**CONTEXT**

**Key Objective**
What is the feasibility to conduct locally relevant breast cancer research at the Uganda Cancer Institute, the national comprehensive cancer center in Uganda?
**Knowledge Generated**
The use of widely available polymerase chain reaction (PCR)–based molecular technology to improve the diagnosis of breast cancer is feasible in Uganda. The test characteristics of reverse transcriptase polymerase chain reaction (RT-PCR) are favorable when compared with the gold standard (immunohistochemistry).
**Relevance**
Given the costs, infrastructure, and trained personnel associated with immunohistochemistry, RT-PCR provides an expedited and cost-effective method to determine receptor testing in breast cancer. Clinical trials conducted in the local setting are both feasible and critical to determining optimal strategies for effective cancer management in Uganda and likely other resource-limited regions. The increased availability of diagnostic tools is one method to reduce the morbidity and mortality of breast cancer in Uganda and elsewhere.

The Uganda Cancer Institute (UCI), the sole national comprehensive cancer center in Uganda, has a long and rich history of clinical investigation and locally relevant cancer research, with an aim of reducing these health disparities. Cancer research in Uganda dates back to the work of Sir Albert Cook and the subsequent research of Sir Denis Burkitt, which helped elucidate the pathogenesis and role of viral oncogenesis and revolutionized the role of combination chemotherapy globally.^9-13^ Burkitt Lymphoma, one of the virus-associated cancers eponymously named after Dr Denis Burkitt, was first described by researchers in Uganda.^14^ Among many other achievements, researchers at the UCI have described the epidemiology of hepatocellular carcinoma, including documenting the relative young age of patients, as well as the presence of advanced disease, when compared with patients in Western Europe and North America, and the benefit of doxorubicin in this setting.^15^ Given the unavailability of radiation therapy in Uganda at the time, Olweny et al^16^ demonstrated the role of combination chemotherapy in pediatric Hodgkin's disease. Indeed, the motto of the UCI is “Research is our resource” to highlight the important role of research in managing patients with cancer.

More recently, given the dual burden of HIV and cancer, colleagues at the UCI recognized the need for less myelotoxic chemotherapeutic options and more pragmatic treatment options among patients with HIV-associated malignancies. Consequently, a prospective study of treatment of two cycles (every 6 weeks) of an oral regimen consisting of lomustine (day 1), etoposide (days 1-3), cyclophosphamide (days 22-26), and procarbazine (days 22-26) was used for patients with HIV-associated non-Hodgkin's lymphoma. In this study, we found that a dose-modified oral regimen of combination chemotherapy was effective and safe with a low rate of treatment-related mortality.^17^ Based on these findings, a randomized clinical trial comparing this oral regimen with parenteral therapy with cyclophosphamide intravenously (IV) on day 1, doxorubicin hydrochloride IV on day 1, vincristine sulfate IV on day 1, and prednisone orally on days 1-5 was launched in SSA (randomized, phase II trial of CHOP vs. oral chemotherapy with concomitant antiretroviral therapy in patients with HIV-associated lymphoma in sub-Saharan Africa; ClinicalTrials.gov identifier: NCT01775475). The results from this multicenter study are forthcoming and importantly demonstrate the role of locally relevant research with widespread implications.

Although HIV-associated malignancies are disproportionately represented in SSA, breast cancer remains among the most common cancers in SSA where it is characterized by advanced stage at diagnosis and poor survival.^18-20^ Despite the increasing investment in cancer care in Uganda and other countries in SSA, survival rates for breast cancer are still markedly lower than those in resource-rich countries.^18^

The Breast Health Global Initiative (BHGI) and the National Comprehensive Cancer Network (NCCN) have published resource-stratified diagnostic and treatment guidelines for low- and middle-income countries. Even in limited-resource settings, the BHGI advocates for the determination of estrogen receptor (ER) status via immunohistochemistry (IHC).^21-24^ Until recently, access to key cancer diagnostic services including locally available diagnostics to detect the presence of hormone receptors (ER and progesterone receptor [PR]) and human epidermal growth factor receptor 2 [HER2] was not readily available in Uganda.

Treatment for breast cancer in Uganda primarily consists of cytotoxic, parenteral therapy and includes cyclophosphamide, methotrexate, and 5-flurouracil (cyclophosphamide, methotrexate, and fluorouracil [CMF]) given IV on days 1 and 8 of a 28-day cycle or cyclophosphamide (orally days 1-14), doxorubicin (IV days 1 and 8), and 5-fluorouracil (days 1 and 8) (cyclophosphamide, doxorubicin, and fluorouracil) administered either every 21 or 28 days. Additionally, a regimen of IV doxorubicin and cyclophosphamide given every 3 weeks followed by a taxane (paclitaxel or docetaxel) has recently come into use in early-stage disease. Each of these regimens are consistent with guidance from the NCCN harmonized guidelines.^25^ As illustrated by our previous work on AIDS-related non-Hodgkin's lymphoma and the need for locally informed clinical decision making, an all-oral adjuvant chemotherapeutic regimen for breast cancer may be more practical than parenteral therapy given the limited availability of central intravenous access, the severe toxicities, and increased costs and logistics associated with IV drug delivery.^17,26^

Over a decade ago, the UCI entered a partnership with the Fred Hutchinson Cancer Research Center (Seattle, WA) to strengthen training and cancer research in Uganda. The mission of this collaboration is to strengthen research capacity, conduct research to reduce the burden of cancer-related morbidity and mortality, and train the next generation of Ugandan clinical researchers.^27^ The UCI, which cares for up to 6,000 patients with newly diagnosed cancer annually, including approximately 700 women with breast cancer, provides a unique opportunity to answer locally relevant questions on the diagnosis and treatment of women with breast cancer. Through the collaboration between the UCI and the Fred Hutch Cancer Research Center, we have conducted over 25 research projects enrolling over 7,000 patients with a nearly 90% participation rate.

Building on this collaboration and with support from GlaxoSmithKline's Africa Non-Communicable Disease Open Lab, we initiated a breast cancer clinical trial with both diagnostic and treatment components at the UCI. Specifically, we aimed to evaluate the potential to use widely available PCR-based molecular technology to improve the diagnosis of breast cancer, and additionally, we designed a companion trial to determine the feasibility of an oral neoadjuvant treatment regimen, consisting of cyclophosphamide, methotrexate, and capecitabine, among Ugandan women with locally advanced breast cancer. In this paper, we will focus on the collaborative efforts of the diagnostic clinical trial, preliminary results, and lessons learned, all of which, we believe, will be of relevance and importance to colleagues engaged in clinical trials in SSA.

## METHODS

For the diagnostic component of this ongoing study, all women > 18 years of age, independent of clinical stage, undergoing a diagnostic assessment for suspected breast cancer at either Mulago Hospital or the UCI are eligible for screening. After informed consent is obtained (in either English or Luganda), the surgeon obtains a biopsy specimen. Specimen collection, preparation, and processing are performed using standard operating procedures, which specify the accurate identification and gross description of the specimen and the prompt transfer of specimens from the surgical suite to the pathology lab. Specimens are divided and processed (fresh frozen and formalin-fixed paraffin-embedded). Only those patients with a histologically confirmed diagnosis of breast cancer are subsequently enrolled in the diagnostic study.

A formalin-fixed paraffin-embedded specimen is used for quantitative RT-PCR, using a validated assay for the detection of the ER, PR, and HER2. Receptor expression levels measured by RT-PCR are expressed as relative quantity (RQ) compared with housekeeping genes (CALM2). The RQ was therefore analyzed for any changes in gene expression in each individual sample relative to the reference gene. The RQ ranged between 0 and 1 with higher values consistent with higher expression of the respective receptors. The RQ cutoff points for each receptor were prespecified by a validated primer set (QuantiTect; Quiagen, Hilden, Germany).

Additionally, unstained slides are sent to the Fred Hutchinson Cancer Research Center to perform IHC for ER, PR, and HER2. The HER2 IHC results are dichotomized as negative (score 0 or 1 + or 2 +) or positive (3 +). The receiver operating characteristic analysis is applied to compare RT-PCR with IHC (the gold standard).

All studies performed under the auspices of this collaboration receive regulatory approval from the institutional review boards at the Fred Hutchinson Cancer Research Center UCI, the Uganda National Council for Science and Technology, and the Uganda Presidents Research Office in a sequential manner.

## PRELIMINARY RESULTS

We analyzed interim data (anticipated N = 100 of an ongoing study) from 32 women of age 35-56 years (median, 48) who participated in the diagnostic component of the study.^28^ The majority of women (25, 78%) presented with advanced stage disease. Of the 32 cancers, 18 were ER+ (56%), 10 were PR+ (31%), and 9 were HER2+ (28%) per IHC; 8 (25%) were triple negative. From receiver operating characteristic analysis, the areas under the curve (95% CI) were 0.94 (0.85 to 1.0), 0.95 (0.89 to 1.0), and 0.76 (0.58 to 0.94) for ER, PR, and HER2, respectively. Selected sensitivity and specificity were 89% and 100% for ER (RQ ≥ 0.085), 100% and 86% for PR (RQ ≥ 0.0019), and 60% and 100% for HER2 (RQ ≥ 0.36), respectively (Table 1).^28^

**TABLE 1:**
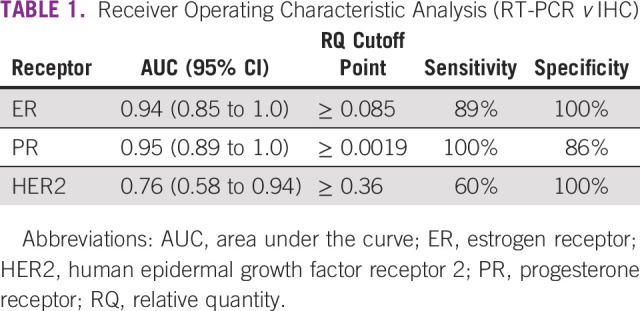
Receiver Operating Characteristic Analysis (RT-PCR *v* IHC)

The study was suspended to accrual secondary to restrictions in place for COVID-19. We anticipate resuming study accrual once the COVID-19 restrictions are lifted.

## DISCUSSION

Beginning with the seminal observations from Sir Albert Cook and Sir Denis Burkit, clinical cancer research in Uganda has been locally relevant and pragmatic and has improved the standard of care of patients living in Uganda and globally. The development of the UCI was facilitated, in part, so that clinical trials could be conducted locally and the landscape of cancer clinical trials in Uganda remains robust. A recent paper by Gopal^29^ reviewed the critical features of cancer clinical trials in Africa, including the need to address a malignancy of high burden locally and the ability to generate novel data specific to the region (but that may be applied more broadly), which yield a favorable cost-benefit ratio. Our current research on breast cancer is in keeping with this mission.

Ideally, cancer clinical trials should be entirely conducted in Uganda and elsewhere in SSA. This current study used the laboratory research infrastructure of both the UCI and the Fred Hutchinson Cancer Research Center. Given the need to determine the test characteristics of a novel method, comparison with a gold standard was necessary. We envision that future similar studies can entirely be conducted locally; however, given the need to compare the PCR results with IHC and the lack of widespread availability of IHC in Uganda at the time of study initiation, such a laboratory collaboration was necessary.

Our study was motivated by both the need for descriptive data on the local prevalence of ER, PR, and HER2 positivity in tumors among Ugandan women with breast cancer and the need to improve diagnostic and treatment options.

A systematic review, which assessed 26 studies from SSA and incorporating nearly 5,000 women, found vast heterogeneity with the majority of estimates of ER-positive disease ranging from 20% to 70%.^7^ Only two small studies were from Uganda, and the data regarding receptor status in Uganda have been mixed. In a study of 65 patients, researchers found that nearly two thirds of samples lacked ER expression; a subsequent study of 45 patients revealed that ER was positive in 60% of specimens.^30,31^ Using IHC in determining breast cancer receptor status is resource-intensive and requires expensive reagents, equipment, and trained personnel. Additionally, the preanalytic handling of the tumor specimen can highly affect results, causing false negative results from mishandling of the specimen and improper fixation time. An accurate determination of receptor status is essential to guide therapeutic decision making, yet this information has not been reliably available in many parts of SSA. Although additional validation is necessary (including the completion of our trial), our preliminary results indicate that the test characteristics of RT-PCR using a platform readily available throughout SSA are promising. Although there are variations in cost, we estimate that the costs associated with RT-PCR are less than half of the costs associated with IHC in the detection of ER, PR, and HER2. Although the laboratory technician time is comparable for both diagnostic tests, RT-PCR precludes much of the specimen handling and processing costs associated with IHC. Additionally, using RT-PCR allows for batching of samples in a cost-efficient manner. Given the wide accessibility of RT-PCR and endocrine therapy for breast cancer in SSA, as well as the recent inclusion of trastuzumab in the WHO's Essential Medicines List, accurately determining breast cancer receptor status has both prognostic and therapeutic implications.

Clearly, increased availability and accessibility of diagnostic measures is necessary but insufficient to reduce the morbidity and mortality of breast cancer in SSA. In a study of 188 patients with breast cancer in Nigeria, 80% of women did not complete chemotherapy in part because of unavailability of drugs and the lack of transportation to the treatment facility.^32^ As such, other options, including effective oral regimens that have the potential to remove some of the barriers to care, are necessary.

In the midst of this study, the severe acute respiratory syndrome coronavirus 2, a novel coronavirus first identified in Wuhan, China, in December 2019 and the agent of the global COVID-19 pandemic emerged.^33^ To curb the transmission of severe acute respiratory syndrome coronavirus 2, the Government of Uganda instituted physical distancing measures and restrictions on the new enrollment into clinical trials (similar measures were in place in Seattle, WA). Patients who were enrolled on the interventional trial continued to receive the oral CMF, with some modifications to the scheduling; however, the study was understandably suspended to new participants. The potential role of oral chemotherapy, which precludes the need for visits to an infusion center, is likely more advantageous in the setting of restricted physical mobility. Indeed, colleagues in Seattle have prescribed this oral regimen of CMF during the COVID-19 pandemic, balancing the risks of travel for infusion therapy with the potential benefits of alternative regimens.

Given the multiple barriers to adequate IHC services, including cost, specimen handling, interpretation, and scarcity of trained pathologists, alternate diagnostic methods are needed. Although preliminary, our data suggest that RT-PCR, which is widely available in Uganda and other countries in SSA, compares favorably with IHC and can inform breast cancer treatment decision making. Patients with tumors expressing ER are typically treated with 5-10 years of endocrine therapy (eg, tamoxifen and aromatase inhibitors) to further reduce the risk of disease recurrence.^34^ Reliable ER testing allows withholding of endocrine therapy (and its associated cost and toxicities) in an ER-negative patient. Patients with tumors expressing HER2 derive benefit from adjuvant HER2-targeted therapy (eg, trastuzumab) for 1 year, although shorter treatment durations may also be beneficial.^35^ However, despite being on the WHO Model List of Essential Medicines, HER2-targeted therapy is not readily available or affordable in resource-limited settings.^36^ Fortunately, biosimilar agents appear to be equally effective and we expect accessibility and availability for these agents to increase in resource-limited regions.^37^ Notably, each of the drugs in the oral CMF regimen are on the List of Essential Medicines, readily available, and recommended by both the National Comprehensive Cancer Network (NCCN) and the BHGI.^38,39^ Although the accrual to the modified CMF clinical trial has been slow, we remain optimistic about the utility of oral chemotherapy in this setting.

Innovative diagnostic and treatment strategies are required to address the burden of breast cancer that is increasing throughout SSA. Given the costs, infrastructure, and trained personnel associated with IHC, alternative testing options (including RT-PCR as tested in our study) may provide an expedited and cost-effective method to determine receptor testing in breast cancer. Alternative treatment options that can reduce the requirement for travel to the infusion center, and potentially reduce the need for costly supportive care medications, are also worthy of exploration. Clinical trials conducted in the local setting are critical to determining optimal strategies for effective breast cancer management in SSA.
